# Age as a key predictor of 6-week mortality in cirrhotic patients with acute gastrointestinal bleeding: a retrospective cohort study

**DOI:** 10.3389/fmed.2026.1709816

**Published:** 2026-01-26

**Authors:** Qi Li, Ruifeng Liu, Shenghui Zhou, Lingna Lyu, Chunlei Fan, Huiguo Ding

**Affiliations:** 1 Department of Gastroenterology and Hepatology, Laboratory for Clinical Medicine, Beijing You’an Hospital, Capital Medical University, Beijing, China; 2 Department of Cardiology, Beijing Friendship Hospital, Capital Medical University, Beijing, China

**Keywords:** acute gastrointestional bleeding, age, intensive care unit, liver cirrhosis, mortality, prognosis

## Abstract

**Background:**

Acute gastrointestinal bleeding (AGIB) in patients with liver cirrhosis is a frequent and often fatal event. This study aimed to thoroughly characterize the relationship between patients’ age and 6-week mortality. We sought to identify specific risk thresholds and key modifying factors to refine clinical risk stratification.

**Methods:**

We conducted a retrospective analysis of 878 patients with liver cirrhosis and AGIB admitted to the Emergency Room at Beijing You’an Hospital. Patients were stratified into age-based tertiles for descriptive analysis. To assess the association between age and 6-week mortality, we built three sequential logistic regression models adjusting for key clinical confounders including the Glasgow-Blatchford Score (GBS), using restricted cubic splines (RCS) to capture non-linear effects and identify risk thresholds. Subgroup analyses and formal tests for interaction were performed to evaluate the consistency of the age-related risk across different clinical scenarios.

**Results:**

The 6-week mortality rate was highest in the oldest age tertile (18.21%). Age emerged as a significant and independent predictor of mortality in all models. The fully adjusted RCS model identified a critical age threshold of approximately 58 years, above which mortality risk increased sharply. The prognostic impact of age was particularly pronounced in male patients and those not receiving endoscopic therapy. Notably, a significant interaction was detected between age and intensive care unit (ICU) admission status (*P* for interaction < 0.05). The strong association between increasing age and higher mortality observed in non-ICU patients was attenuated and no longer significant in those admitted to the ICU. A significant association between increasing age and 6-week mortality was identified in patients with Child-Pugh grade C (*p* < 0.001), and in medium-risk and high-risk groups (both *p* = 0.011) when patients were stratified based on GBS. Additionally, in the etiological subgroups, age was a significant predictor of 6-week mortality only in patients with viral cirrhosis (*p* = 0.002) and viral/alcoholic cirrhosis (*p* = 0.01), but not in patients with other etiologies.

**Conclusion:**

Age is a critical independent predictor of 6-week mortality in cirrhotic patients with AGIB, but its prognostic effect varies with the level of care. Specifically, It strongly predicts mortality in non-ICU settings, but not in the ICU. This challenges the uniform view of age as a risk factor and suggests that early transfer to higher-level care such as ICU admission may reduce age-related risk in this vulnerable population.

## Introduction

Acute gastrointestinal bleeding (AGIB) remains a formidable challenge in gastroenterology and hepatology. It is a life-threatening emergency associated with substantial morbidity and mortality, particularly in patients with liver cirrhosis (1, 2). The intricate pathophysiology of cirrhosis including coagulopathy, portal hypertension, and a diminished physiological reserve place patients at high risk of rapid decompensation and death following a bleeding event (3, 4). Consequently, the ability to accurately stratify risk upon a patient’s initial presentation is paramount for guiding management strategies and improving survival.

A growing body of evidence has established patients’ age as a significant predictor of mortality in AGIB (5, 6). Moreover, advanced age has been consistently linked to worse outcomes including higher mortality rates. Recent large-scale machine learning studies have confirmed that age is one of the most powerful predictors of in-hospital mortality among cirrhotic patients with AGIB (7).

Although this is recognized, important gaps in our understanding of age-related mortality risk still remain. First, specific age thresholds that might indicate a sharp risk increase have not been clearly defined for this population. This limits the translation of knowledge into practical clinical guidelines. Second, and perhaps more importantly, it remains largely unclear how the relationship between age and mortality is influenced or modified by crucial clinical factors. These include the presence of comorbidities, the application of specific treatments such as endoscopy and the intensity of care provided (i.e., general ward and Intensive Care Unit (ICU) admission). This lack of detailed understanding hinders the development of precise clinical guidelines tailored to different age groups.

Accordingly, this study was designed to address these gaps. We sought to thoroughly investigate the relationship between age and 6-week mortality rate in a large, well-characterized cohort of cirrhotic patients with AGIB. Our primary objectives were to move beyond simple associations by (1) identifying clinically relevant age thresholds for mortality risk, and (2) exploring how this relationship is modified by other key clinical variables, with a particular focus on the pivotal interaction with ICU admission.

## Methods

### Study design and patient population

This retrospective cohort study was conducted at the Emergency Department of Beijing You’an Hospital, a tertiary referral center for liver diseases. We reviewed the electronic medical records of all adult patients (aged ≥18 years) who were admitted for a primary diagnosis of AGIB between April 1st 2021 and December 31st 2022. For patients with multiple admissions during the study period, only data from the first admission were included in the analysis. The study enrolled patients with a confirmed diagnosis of liver cirrhosis based on clinical, histological or imaging criteria. We excluded patients who were: (i) discharged against medical advice from the Emergency Department; (ii) lost to follow-up within the 6-week study period; (iii) diagnosed with concurrent hepatocellular carcinoma (HCC) or other malignancies; (iv) previously diagnosed with chronic kidney disease. After applying these criteria, 878 patients were eligible and followed up for 6 weeks. The primary endpoint for this study was all-cause mortality within 6 weeks of admission. All patients were divided into three groups based on the age percentile grouping method.

### Data collection and definitions

We extracted baseline demographic data, clinical characteristics, laboratory parameters, and treatment information from the hospital’s electronic records between April 1^st^ 2021 and December 31^st^ 2022. The diagnosis of liver cirrhosis was established based on a combination of medical history, physical examination, characteristic laboratory parameters, and imaging studies (ultrasound, CT, or MRI), according to the established Chinese guideline (8). AGIB was defined as hematemesis, melena, or hematochezia originating from the gastrointestinal tract. HCC was diagnosed according to Chinese national guidelines (9).

Patients were admitted to the ICU based on institutional guidelines, which include the presence of one or more of the following: (1) hemodynamic instability (e.g., systolic blood pressure < 90 mmHg) refractory to initial fluid resuscitation; (2) need for mechanical ventilation; (3) massive and ongoing hematemesis or hematochezia requiring intensive monitoring; (4) altered mental status (hepatic encephalopathy Grade III-IV or a Glasgow Coma Scale score < 9); or (5) the requirement for vasopressor support to maintain mean arterial pressure.

The Glasgow-Blatchford score (GBS) was calculated based on patients’ vital signs, including systolic blood pressure and heart rate, laboratory data such as blood urea nitrogen (BUN) and hemoglobin (Hb), and medical history including hepatic disease and heart failure. Each item was assigned a score ranging from 0 to 6 points, with the total score ranging from 0 to 23 points. The total points corresponded to the patients’ risk category. Patients with a GBS score less than 6 points were stratified as a low risk group, those with a GBS score of 12 points or more as a high risk group, and patients with a GBS score between 6 and 11 points were classified in the medium risk group.

### Statistical analysis

Missing data for covariates were handled using multiple imputation with five imputations. Continuous variables were presented as the median and interquartile range (IQR) and compared using the Mann–Whitney U test or Kruskal-Wallis test, as appropriate. Categorical variables were reported as frequencies and percentages (%) and compared using the Chi-square test or Fisher’s exact test. To model the association between age and 6-week mortality, we constructed three sequential logistic regression models, with age entered as a continuous variable: Model 1, a univariable model, assessed the crude association between age (as a continuous variable) and the primary outcome. Model 2 was adjusted for gender to account for fundamental demographic confounding. Model 3 was the fully adjusted multivariable model, which included the variables from Model 2 plus a comprehensive set of clinically relevant covariates. These were selected to control for disease severity, baseline hemodynamics, lifestyle factors, and major comorbidities, and included the GBS, systolic and diastolic blood pressure on admission, smoking history, alcohol consumption, hypertension, diabetes, coronary heart disease, BUN, and creatinine (Cr). Candidate variables for this model were selected based on clinical importance and statistical significance (*p* < 0.10) in univariable analyses. The final model was determined using a backward stepwise selection algorithm. Within each model, we employed restricted cubic splines (RCS) with four knots to flexibly model and visualize the potential non-linear dose–response relationship. The age at which the odds ratio (OR) for mortality crossed from below to above 1.0 was identified as the critical risk threshold. Subgroup analyses were performed to evaluate the consistency of the age-mortality association across various clinical strata, including Child-Pugh grade, GBS and etiology of liver cirrhosis. Furthermore, a formal test for interaction was conducted by including a multiplicative interaction term (e.g., age × ICU-admission) in the multivariable logistic regression model. All statistical tests were two-sided with *p* values < 0.05 considered statistically significant. Analyses were performed using R software, version 4.2.1 (R Foundation for Statistical Computing, Vienna, Austria).

## Results

### Baseline characteristics of the study cohort

**Figure 1:**
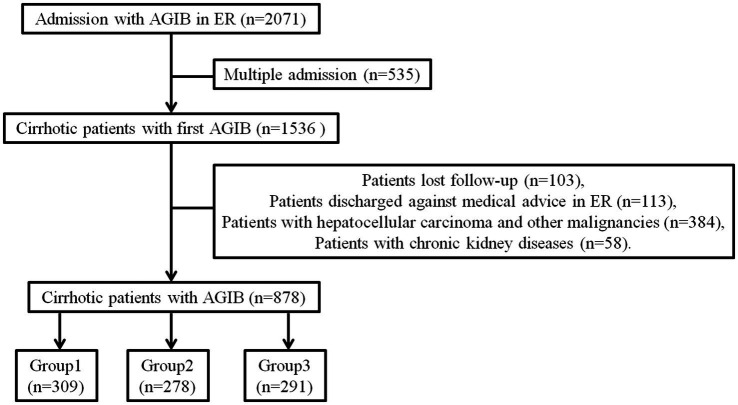
Schematic diagram of patient selection procedure.

A total of 2,071 electronic records of patients with emergent AGIB were reviewed. Patients with multiple admissions were excluded to avoid duplicate data, and screening was conducted according to the inclusion/exclusion criteria. Ultimately 878 cirrhotic patients with AGIB were enrolled and followed up for 6 weeks (Figure 1). The median age of the cohort was 58 years (IQR 50–65), with males comprising 66.63% of the cohort. The overall 6-week mortality rate for the cohort was 12.87% (113 out of 878 patients).

When all patients were stratified by age into tertiles, significant differences in baseline characteristics and outcomes were observed (Table 1). There were 309 patients in Group 1 with a median age of 47 years; 278 in Group 2 with a median age of 58 years; and 291 in Group 3 with a median age of 68 years. The 6-week mortality rate rose progressively with age, from 10.03% in the youngest group (Group 1) to 10.43% in the middle group (Group 2), and peaked at 18.21% in the oldest group (Group 3, *P* for trend < 0.001). Patients in the oldest group (Group 3) had a significantly higher prevalence of comorbidities, including hypertension and coronary heart disease, but paradoxically had lower rates of prior smoking and alcohol consumption. Additionally, they had lower rates of portal vein thrombosis (PVT) and liver failure. There was a notable demographic shift characterized by a decreasing proportion of male patients as age increased. Importantly, there were no significant differences among the groups in terms of presenting bleeding symptoms, need for ICU admission, or baseline albumin, Hb, Cr, and TnI levels. In contrast, the oldest group (Group 3) had the lowest levels of TB, PT, ALT, AST and INR, but the highest levels of PLT, BUN, CK-MB, and myoglobin among the three groups.

**Table 1:** Clinical characteristics of patients with cirrhotic AGIB in distinct age groups.

Variables	All (*n* = 878)	Group1 (*n* = 309)	Group2 (*n* = 278)	Group3 (*n* = 291)	*P*
Age, (median [IQR])	58.00 (50.00, 65.00)	47.00 (40.00, 50.00)	58.00 (56.00, 60.00)	68.00 (65.50, 74.00)	<0.001
Male, *n* (%)	585 (66.63%)	268 (86.73%)	191 (68.71%)	126 (43.30%)	<0.001
Melena, *n* (%)	504 (57.40%)	172 (55.66%)	168 (60.43%)	164 (56.36%)	0.459
Hematemesis, *n* (%)	589 (67.08%)	213 (68.93%)	183 (65.83%)	193 (66.32%)	0.686
Hematochezia, *n* (%)	80 (9.11%)	33 (10.68%)	22 (7.91%)	25 (8.59%)	0.474
Systolic blood pressure on admission (mmHg)	117.00 (104.00, 132.00)	115.00 (102.00, 129.00)	118.90 (105.00, 135.00)	118.00 (104.00, 135.00)	0.080
Diastolic blood pressure on admission (mmHg)	67.00 (59.00, 75.00)	68.00 (61.00, 76.00)	68.00 (60.00, 77.00)	65.00 (58.00, 74.00)	0.011
Shock index, (median [IQR])	0.76 (0.65, 0.88)	0.79 (0.70, 0.92)	0.75 (0.63, 0.85)	0.74 (0.64, 0.86)	<0.001
Hypertension, *n* (%)	19 2(21.87%)	31 (10.03%)	63 (22.66%)	98 (33.68%)	<0.001
Diabetes, *n* (%)	237 (26.99%)	62 (20.06%)	87 (31.29%)	88 (30.24%)	0.003
Coronary heart disease, *n* (%)	49 (5.58%)	4 (1.29%)	15 (5.40%)	30 (10.31%)	<0.001
Smoking history, *n* (%)	327 (37.24%)	151 (48.87%)	111 (39.93%)	65 (22.34%)	<0.001
Drinking history, n (%)	419(47.72%)	189(61.17%)	142(51.08%)	88(30.24%)	<0.001
Liver failure, *n* (%)	101(11.50%)	50(16.18%)	27(9.71%)	24(8.25%)	0.005
First bleeding, *n* (%)	375 (42.71%)	138 (44.66%)	106 (38.13%)	131 (45.02%)	0.174
Endoscopy treatment, *n* (%)	429(48.86%)	141(45.63%)	155(55.76%)	133(45.70%)	0.021
PVT, n (%)	285 (32.46%)	90 (29.13%)	111(39.93%)	84(28.87%)	0.006
Length of hospitalization, (d)	10.00 (7.00, 14.00)	10.00 (6.00, 14.00)	11.00 (7.00, 14.00)	10.00 (6.00, 14.00)	0.340
6-week mortality (%)	113 (12.87%)	31 (10.03%)	29 (10.43%)	53 (18.21%)	0.004
Admitted to ICU, *n* (%)	34 (3.87%)	14 (4.53%)	10 (3.60%)	10 (3.44%)	0.754
Child-Pugh score, (median [IQR])	9.00 (8.00, 11.00)	10.00 (8.00, 11.00)	9.00 (7.25, 11.00)	9.00 (7.00, 10.00)	0.001
TB (μmol/L) (median [IQR])	24.40 (15.62, 44.72)	28.50 (17.60, 59.40)	25.00 (15.43, 43.25)	22.10 (13.60, 34.45)	<0.001
PT (s) (median [IQR])	15.60 (13.90, 18.38)	16.60 (14.60, 19.60)	15.55 (13.90, 17.98)	14.70 (13.10, 16.96)	<0.001
Hb, (g/L) (median [IQR])	77.00 (61.00, 95.75)	77.00 (61.00, 99.00)	77.50 (60.25, 94.75)	78.00 (61.00, 93.00)	0.747
Hct (median [IQR])	23.80 (19.20, 29.20)	23.70 (19.40, 30.00)	23.75 (18.80, 28.45)	23.80 (19.30, 28.65)	0.565
PLT (*10^9^/L) (median [IQR])	77.00 (52.00, 112.00)	71.00 (48.00, 108.00)	73.00 (52.00, 110.75)	86.00 (59.00, 121.50)	0.002
BUN (mmol/L) (median [IQR])	9.60 (6.96, 13.11)	9.47 (7.02, 12.15)	8.96 (6.53, 12.78)	10.23 (7.29, 14.83)	0.001
Cr (μmol/L) (median [IQR])	61.00 (50.00, 78.75)	62.00 (51.00, 75.00)	59.00 (50.00, 75.00)	63.00 (50.00, 85.00)	0.169
ALT (U/L) (median [IQR])	27.00 (20.00, 37.00)	29.00 (22.00, 40.00)	28.00 (21.00, 38.75)	23.00 (18.00, 32.00)	<0.001
AST (U/L) (median [IQR])	34.00 (25.00, 55.00)	37.00 (28.00, 61.00)	35.00 (26.00, 57.00)	31.00 (23.00, 44.00)	0.001
CK-MB (ng/ml) (median [IQR])	0.76 (0.48, 1.35)	0.67 (0.43, 1.15)	0.79 (0.51, 1.44)	0.80 (0.50, 1.37)	0.001
Myoglobin (ng/ml) (median [IQR])	44.00 (27.00, 87.00)	41.00 (25.00, 74.00)	44.00 (26.00, 94.75)	48.00 (30.50, 87.50)	0.038
TnI (ng/ml) (median [IQR])	0.05 (0.04, 0.06)	0.05 (0.05, 0.06)	0.05 (0.04, 0.06)	0.05 (0.04, 0.06)	0.496
APTT (s) (median [IQR])	31.50 (28.20, 35.20)	32.00 (28.70, 35.70)	31.34 (28.02, 34.95)	31.10 (28.10, 35.00)	0.205
INR (median [IQR])	1.39 (1.24, 1.64)	1.48 (1.30, 1.74)	1.38 (1.24, 1.60)	1.31 (1.17, 1.51)	<0.001

PVT, Portal vein thrombosis; ICU, Intensive Care Unit; TB, Total bilirubin; PT, Prothrombin time; Hb, Hemoglobin; Hct, Hematocrit; PLT, Platelet; BUN, Blood urea nitrogen; Cr, Creatinine; ALT, Alanine transferase; AST, Aspartate transaminase; CK-MB, Creatinine kinase-MB; TnI, Troponin I; APTT, Activated partial thromboplastin time; INR, International normalized ratio.

### Age as an independent predictor of 6-week mortality

**Figure 2:**
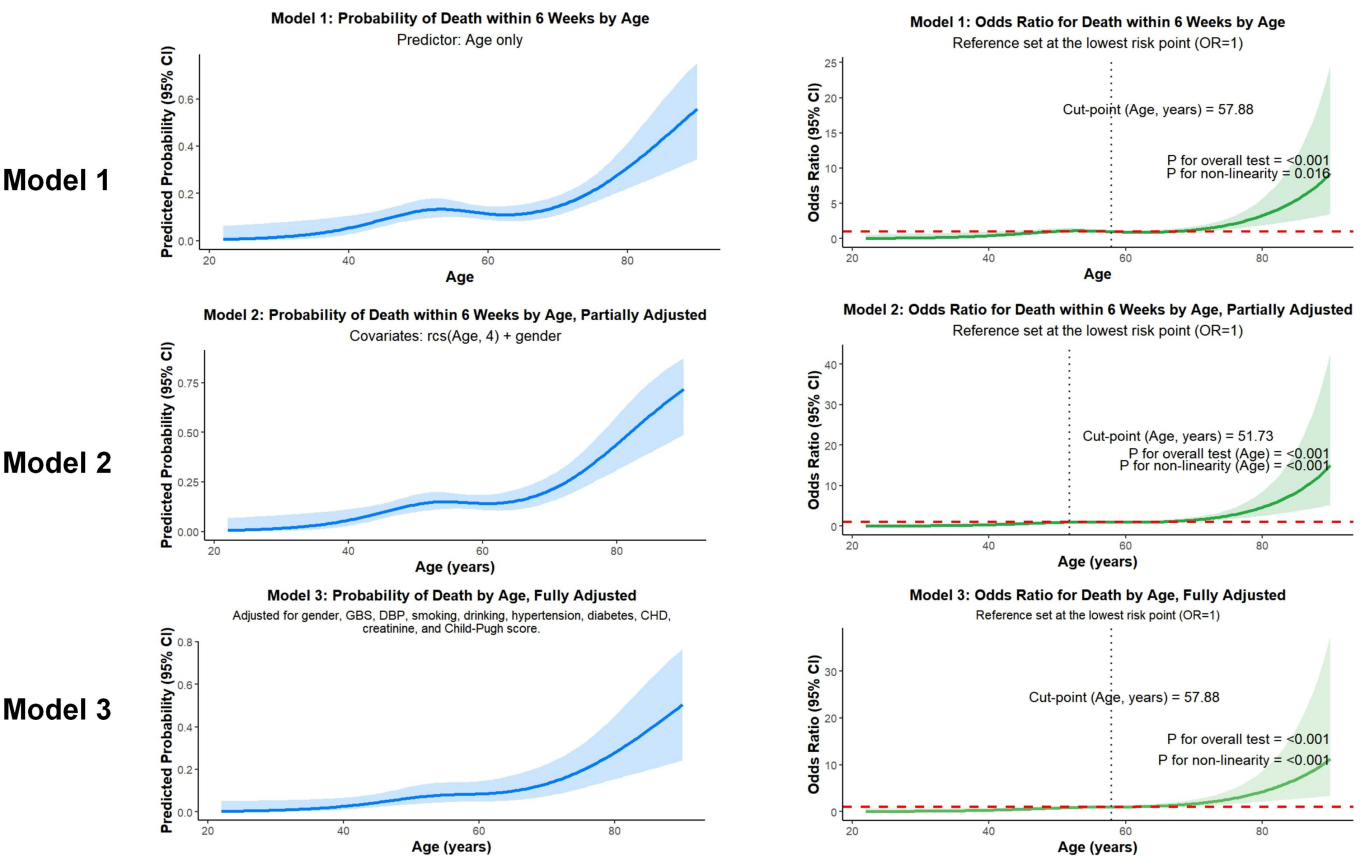
Association of age and 6-week mortality in 3 models.

In our regression analyses, age as a continuous variable was strongly associated with 6-week mortality. This association remained robust and statistically significant after sequential adjustment for gender (Model 2) and a comprehensive set of potential confounders, including patient demographics, baseline vitals, and the GBS in the final multivariable model (Model 3, Figure 2).

**Figure 3:**
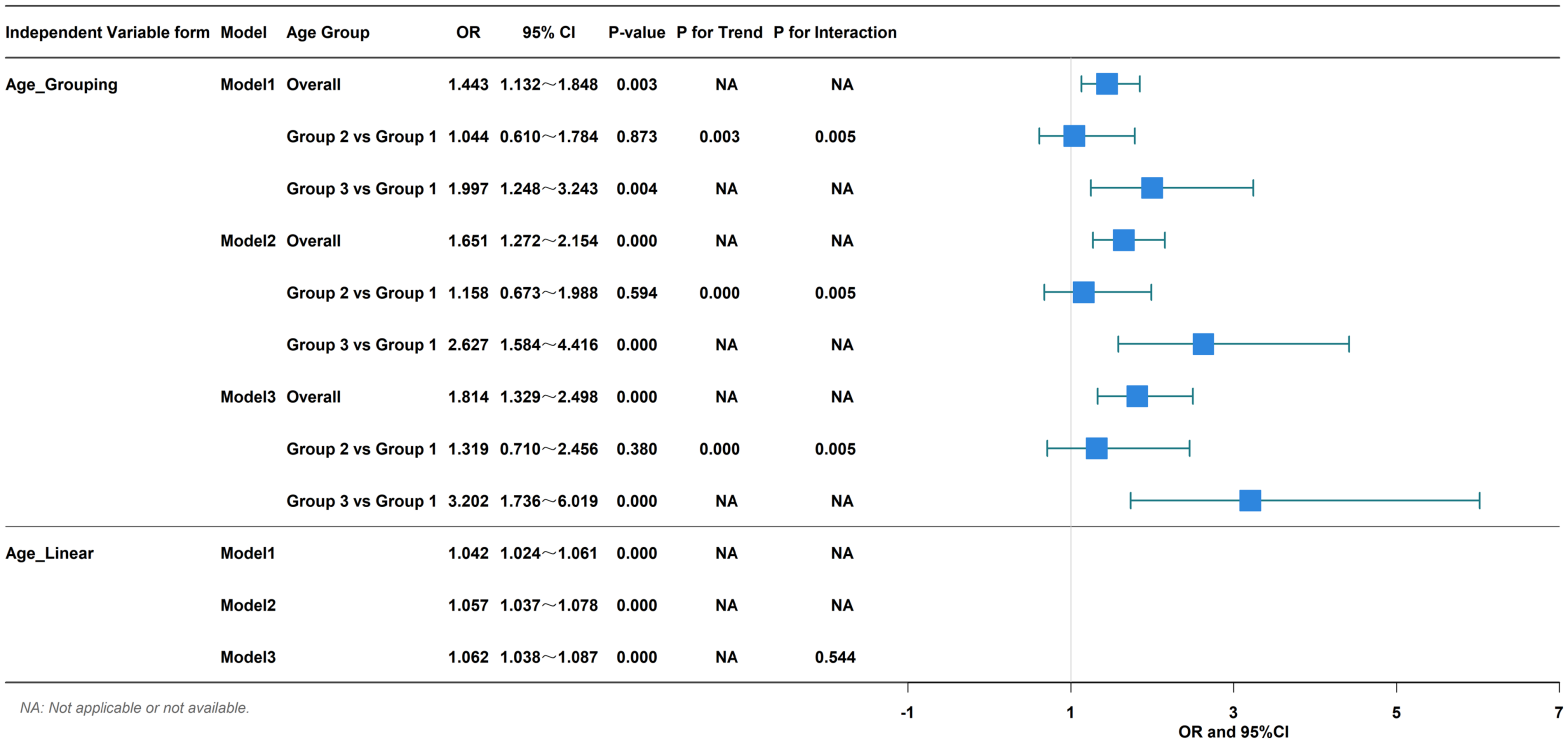
Association of age and 6-week mortality by RCS analysis.

**Figure 4:**
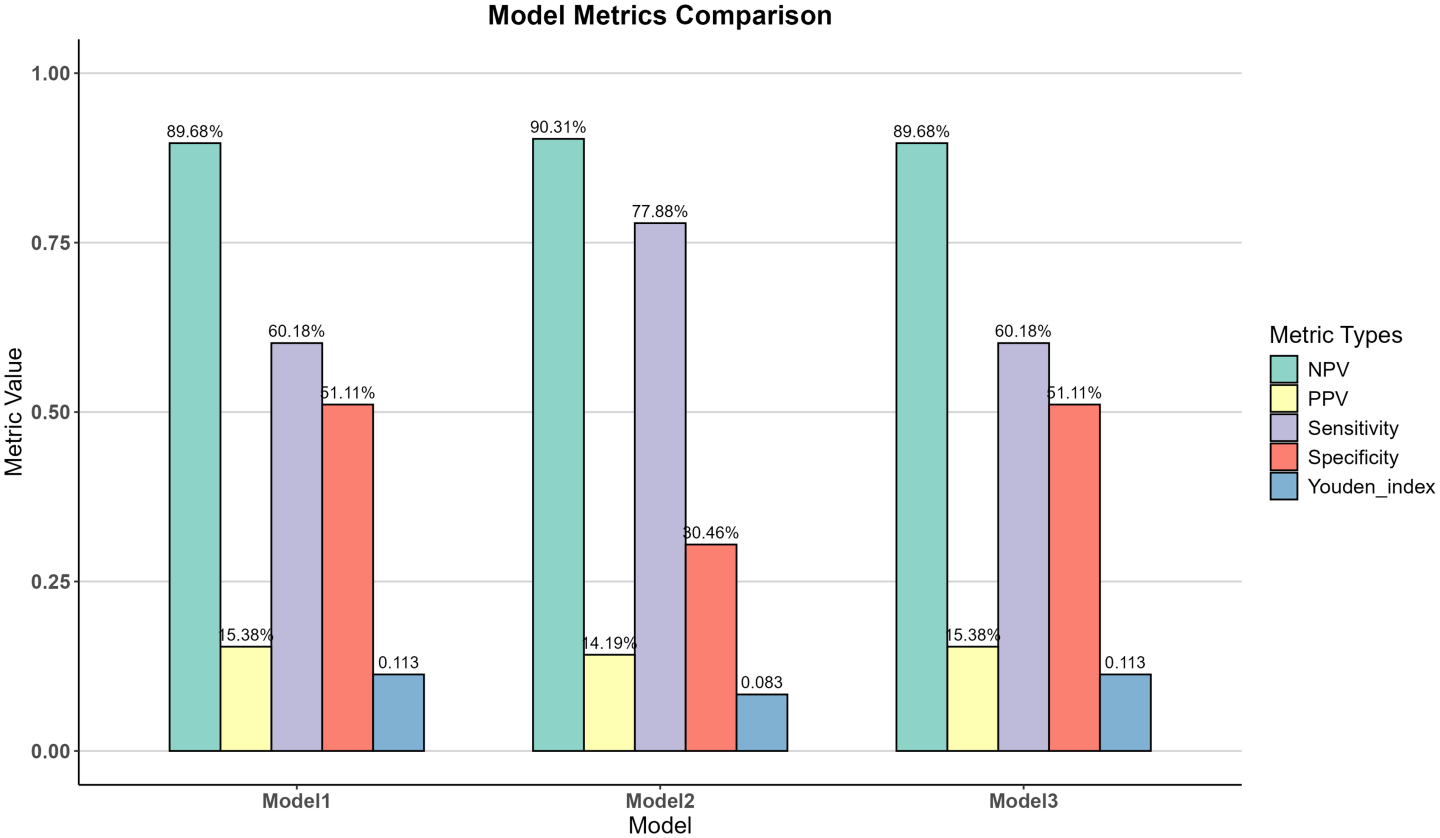
Model metrics comparison among 3 models.

Additionally, a RCS analysis was conducted to model the relationship between age and 6-week mortality. The results revealed a distinct non-linear pattern: the odds of mortality remained relatively low and stable in younger patients before escalating sharply in older individuals (Figure 3). Based on the fully adjusted model (model 3), we identified a critical inflection point at an age of 57.88 years, where the OR for mortality risk began to exceed 1.0 (*p* < 0.001). To evaluate the diagnostic utility of this age threshold, we assessed its performance across different models. This analysis showed that the partially adjusted model (model 2) yielded the highest sensitivity at 77.88%, while the unadjusted model (model 1) and fully adjusted model (model 3) offered the highest specificity at 51.11% (Figure 4).

### Subgroup analyses and the pivotal interaction with ICU admission

**Figure 5:**
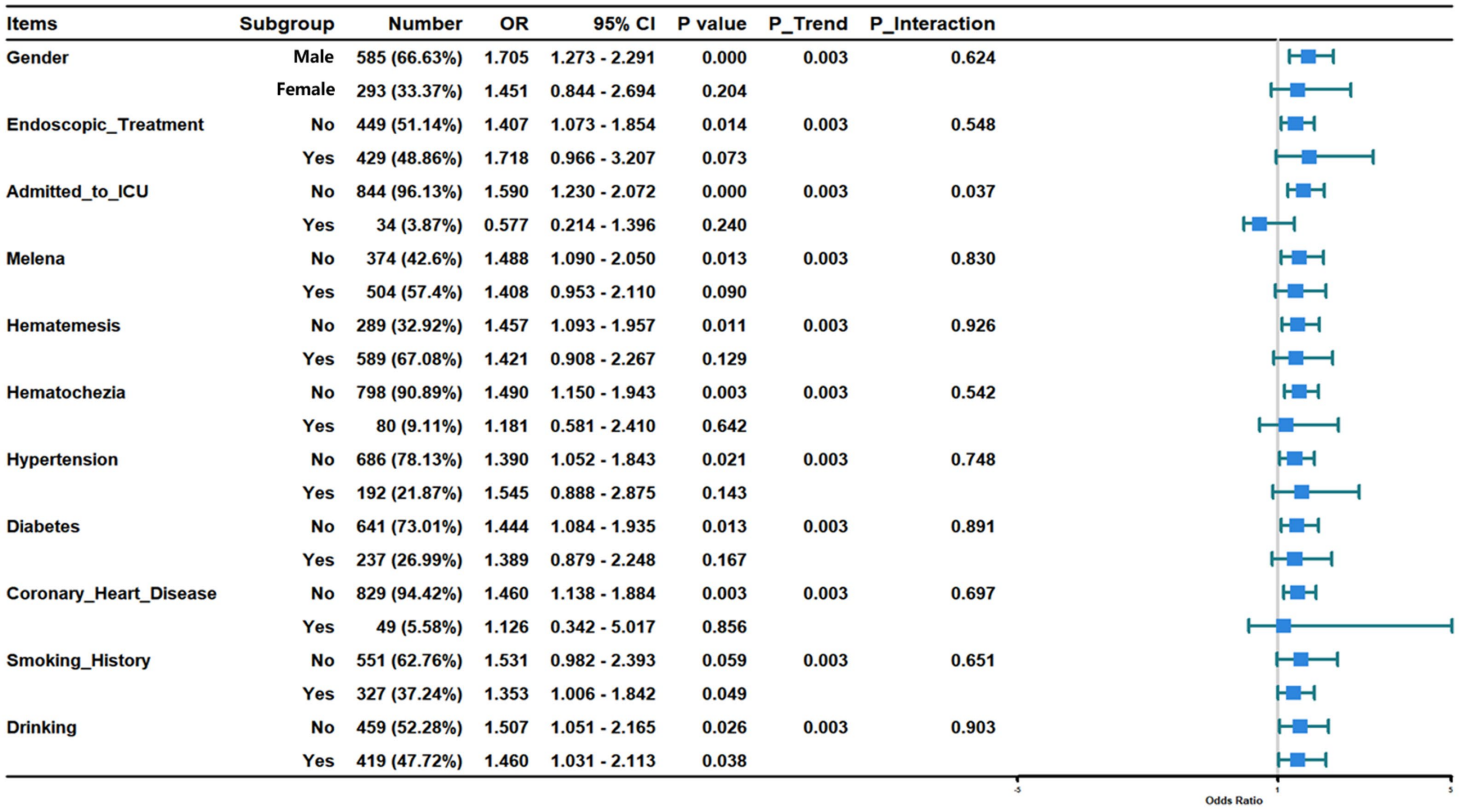
Association of age and 6-week mortality in subgroups.

To understand if the prognostic effect of age was consistent across different patient subsets, we performed extensive subgroup analyses (Figure 5). In male patients, age was associated with 6-week mortality (OR, 1.705; 95%CI, 1.273–2.291; *p* < 0.001), but no association between age and 6-week mortality existed in female patients. For patients who did not receive endoscopic treatment, age was associated with 6-week mortality (OR, 1.407; 95%CI, 1.073–1.854; *p* = 0.014), but there was no association between age and 6-week mortality in patients who received endoscopic treatment. Age was associated with 6-week mortality in patients without symptoms of melena (OR, 1.488; 95%CI, 1.090–2.050; *p* = 0.003), hematemesis (OR, 1.457; 95%CI, 1.093–1.957; *p* = 0.003) and hematochezia (OR, 1.490; 95%CI, 1.150–1.943; *p* = 0.003). In patients with these symptoms, however, there was no relationship between age and 6-week mortality. Regarding comorbidities, age was associated with 6-week mortality in patients without hypertension (OR, 1.390; 95%CI, 1.052–1.843; *p* = 0.021), diabetes (OR, 1.444; 95%CI, 1.084–1.935; *p* = 0.003) and coronary heart disease (OR, 1.460; 95%CI, 1.138–1.884; *p* = 0.003), but in patients with hypertension, diabetes and coronary heart disease, age was not associated with 6-week mortality. In patients with history of smoking, age was associated with 6-week mortality (OR, 1.353; 95%CI, 1.006–1.842; *p* = 0.049), however, age was not associated with 6-week mortality in patients who did not smoke. Age was associated with 6-week mortality regardless of history of alcohol consumption (OR, 1.460; 95%CI, 1.031–2.113; *p* = 0.038; OR, 1.507; 95%CI, 1.051–2.165; *p* = 0.026). In all the above subgroups, the P for interaction was greater than 0.05, indicating that gender, endoscopic treatment, melena, hematemesis, hematochezia, hypertension, diabetes, coronary heart disease, smoking history, and alcohol consumption did not significantly modify the effect of age on mortality.

Notably, a formal test for interaction revealed that the decision to admit a patient to the ICU fundamentally altered the prognostic significance of age (*P* for interaction < 0.05). In the large group of patients who were not admitted to the ICU, increasing age was a powerful predictor of death (OR, 1.590; 95% CI, 1.230–2.072; *p* < 0.001). In stark contrast, among the subset of patients who were admitted to the ICU, age lost its statistical association with mortality. This finding demonstrates that the ICU environment attenuates the typical age-related risk seen in the general ward population.

### Subgroup analysis of the association between age and 6-week mortality based on Child-Pugh grade, GBS and etiology of liver cirrhosis

**Figure 6:**
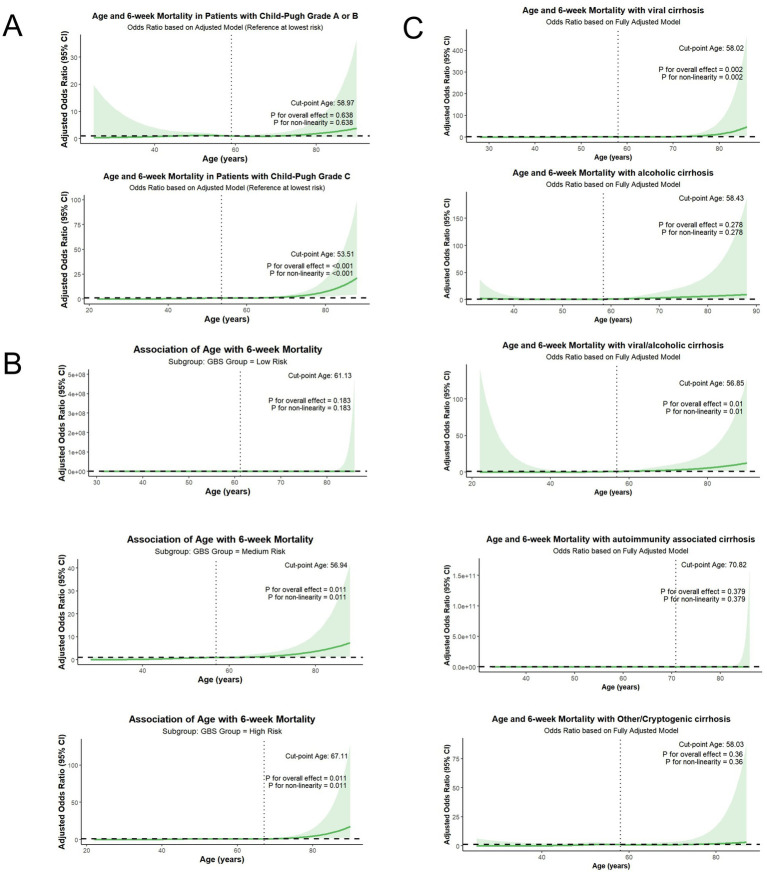
Subgroup analysis of the association between age and 6-week mortality based on Child-Pugh grade, GBS and etiology of liver cirrhosis. **(A)** analysis of the association between age and 6-week mortality based on Child-Pugh grade; **(B)** analysis of the association between age and 6-week mortality based on GBS; **(C)** analysis of the association between age and 6-week mortality based on etiology of liver cirrhosis.

To further evaluate the heterogeneity in the association between age and 6-week mortality, we performed subgroup analyses based on Child-Pugh grade, GBS and etiology of liver cirrhosis (Figure 6). A statistically significant association between increasing age and 6-week mortality was identified in patients with Child-Pugh grade C (*p* < 0.001), with a cut-off age of 53.5 years. However, this association was not statistically significant in patients with Child-Pugh grade A or B (*p* = 0.638) (Figure 6A). Patients were stratified into low-risk, medium-risk and high-risk groups based on GBS, revealing statistically significant associations between age and 6-week mortality in both medium-risk and high-risk groups (both *p* = 0.011), but no significant association was found in the low-risk group (*p* = 0.183) (Figure 6B). Regarding the etiology subgroups, age was a significant predictor of 6-week mortality for patients with viral cirrhosis (*p* = 0.002) and viral/alcoholic cirrhosis (*p* = 0.01). In contrast, no statistically significant association was observed between age and mortality risk in the subgroups of patients with alcoholic cirrhosis, autoimmunity-associated cirrhosis, or other etiologies (*p* = 0.278, *p* = 0.379, and *p* = 0.36, respectively) (Figure 6C).

## Discussion

In this large cohort of cirrhotic patients presenting with AGIB, we confirmed that advancing age is a powerful and independent predictor of 6-week mortality. Our study, however, extends this understanding in three crucial ways. First, we defined a clinically relevant age threshold of approximately 58 years, beyond which mortality risk begins to increase significantly. Second, we identified specific patient subgroups, such as males, patients with Child-Pugh C grade, patients in GBS medium-risk/high-risk groups and patients with viral cirrhosis or viral/alcoholic cirrhosis, in whom this risk is more pronounced. Third, and most importantly, we uncovered a pivotal interaction: the detrimental effect of age on survival clearly. Evident in the general patient population was no longer significant in patients managed within an ICU.

Our primary finding that age is an independent risk factor for mortality in cirrhotic patients with AGIB aligns with and reinforces previous research (7, 10–12). The innovative aspect of our study lies in its focus on the specific impact of age on 6-week mortality in patients suffering from AGIB related to liver cirrhosis. To achieve this, we employed RCS analysis, moving beyond a simple linear assumption to describe a more nuanced, non-linear relationship between age and 6-week mortality in these patients. Our findings indicate distinct age cut-off points for mortality risk, which have not been previously established in the literature. This study identified general trends in mortality across age groups and provided specific cut-off values: 57.88 years, 51.73 years, and 57.88 years, which can serve as preliminary benchmarks for clinicians in emergency settings. This has direct clinical implications. Although age is a continuum, an identified threshold can serve as a valuable “cognitive stop sign” for clinicians in a busy emergency department. It suggests that patients in their late 50s and older warrant a higher level of suspicion for increased mortality risk and potentially more aggressive initial management, irrespective of their initial clinical appearance.

The most novel and clinically significant finding of our study is the significant modifying effect of ICU admission. The observation that age’s prognostic power was nullified in the ICU setting is compelling. This appears to contrast with some studies of heterogeneous cirrhotic ICU populations, where age remained a predictor of death (13, 14). We hypothesize this discrepancy arises from our specific focus on the AGIB context. It is plausible that the aggressive, protocol-driven hemodynamic resuscitation and continuous multi-organ monitoring, along with rapid intervention capabilities inherent to intensive care, can effectively buffer the age-related decline in physiological reserve. In this environment, the patient’s acute illness severity and response to treatment may become the dominant determinants of outcome, overshadowing the influence of chronological age. Conversely, on a general ward an older patient’s underlying frailty and reduced capacity to withstand the prolonged physiological stress of a major bleed may become the decisive factor in their clinical trajectory. This finding strongly suggests that for older cirrhotic patients with AGIB, early consideration for ICU admission may be a critical, outcome-modifying decision.

Our findings also complement recent advancements in prognostic modeling, such as the ‘Score for Cirrhosis with Acute Gastrointestinal Bleeding’ (CAGIB) (15) and its machine learning-based enhancements (16). While these complex multi-component scores offer high predictive accuracy, our study highlights that age, a simple and universally available parameter, not only retains powerful independent prognostic value but also acts as a critical effect modifier based on the level of care. This suggests that future research could explore whether integrating our age threshold and its interaction with ICU status into models like CAGIB could further refine their predictive power, especially for triaging older patients at the point of first contact.

The strengths of our study include its large sample size focused on a high-risk population, a clinically meaningful primary endpoint defined as 6-week mortality, and the use of sophisticated statistical methods to provide a detailed analysis. However, several limitations must be acknowledged. First, the retrospective single-center design may be subject to selection bias and limits the external generalizability of our findings. Moreover, a major limitation is the inability to differentiate between variceal and non-variceal bleeding sources due to limited availability and delayed access to emergent endoscopic services in our setting. Since these conditions have different prognoses, combining them could have diluted specific effects on outcomes. In addition, data on other important prognostic factors were not uniformly available. Specifically, for some patients with a first bleeding episode who did not undergo immediate endoscopy, we could not definitively classify their decompensation status, which is a known limitation. Furthermore, objective measures of physical fitness or frailty were not systematically recorded in the historical patient records, precluding a deeper analysis of the factors underpinning age-related physiological reserve. Future research should address these limitations by conducting multi-center studies with larger sample sizes. Such studies would enhance the robustness of the findings and allow for more comprehensive risk stratification across diverse patient groups. Furthermore, long-term follow-up could provide valuable insights into the persisting effects of age on mortality beyond the initial six-week period. This would contribute to a more nuanced understanding of AGIB management in cirrhotic patients.

In conclusion, our research elucidates the critical role of age as an independent risk factor for 6-week mortality in cirrhotic patients experiencing AGIB. We provide a specific age threshold that can immediately inform clinical risk assessment at the bedside. More importantly, our findings highlight that the decision regarding ICU admission is a powerful modifier of this age-related risk. This underscores the need for clinicians to incorporate age not merely as a static variable, but as a dynamic factor in clinical decision-making, particularly when determining the appropriate level of care and resource allocation for this vulnerable patient population. Future multi-center prospective studies are needed to validate these findings and to explore the mechanisms underlying these important observations.

## Data Availability

The raw data supporting the conclusions of this article will be made available by the authors, without undue reservation.

## References

[1] Stanley AJ Laine L. Management of acute upper gastrointestinal bleeding. BMJ. (2019) 364:l536 30910853 10.1136/bmj.l536

[2] Ibrahim M Mostafa I Devière J. New developments in managing variceal bleeding. Gastroenterology. (2018) 154:1964–9. doi: 10.1053/j.gastro.2018.02.023, 29481777

[3] D'Amico G De Franchis R Cooperative Study Group. Upper digestive bleeding in cirrhosis. Post-therapeutic outcome and prognostic indicators. Hepatology. (2003) 38:599–612. doi: 10.1053/jhep.2003.50385 12939586

[4] Gralnek IM Garcia-Pagan JC Hernández-Gea V. Challenges in the management of esophagogastric varices and variceal hemorrhage in cirrhosis - a narrative review. Am J Med. (2024) 137:210–7. doi: 10.1016/j.amjmed.2023.12.001 38128860

[5] Arai M Matsumura T Ohta Y Kiyono S Hayashi M Taida T . Long-term prognosis of patients with obscure gastrointestinal bleeding: a retrospective cohort study. Digestion. (2019) 100:37–44. doi: 10.1159/000493854 30636251

[6] Lecleire S Di Fiore F Merle V Hervé S Duhamel C Rudelli A . Acute upper gastrointestinal bleeding in patients with liver cirrhosis and in noncirrhotic patients: epidemiology and predictive factors of mortality in a prospective multicenter population-based study. J Clin Gastroenterol. (2005) 39:321–7. 15758627 10.1097/01.mcg.0000155133.50562.c9

[7] Tsai SC Lin CH Chu CCJ Lo HY Ng CJ Hsu CC . Machine learning models for predicting mortality in patients with cirrhosis and acute upper gastrointestinal bleeding at an emergency department: a retrospective cohort study. Diagnostics. (2024) 14:1919. doi: 10.3390/diagnostics14171919, 39272704 PMC11394157

[8] Xu XY Ding HG Li WG Xu JH Han Y Jia JD . Chinese guidelines on the management of liver cirrhosis (abbreviated version). World J Gastroenterol. (2020) 26:7088–103. doi: 10.3748/wjg.v26.i45.7088, 33362370 PMC7723671

[9] Zhou J Sun HC Wang Z Cong WM Wang JH Zeng MS . Guidelines for diagnosis and treatment of primary liver cancer in China (2017 edition). Liver Cancer. (2018) 7:235–60. doi: 10.1159/000488035, 30319983 PMC6167671

[10] del Olmo JA Peña A Serra MA Wassel AH Benages A Rodrigo JM. Predictors of morbidity and mortality after the first episode of upper gastrointestinal bleeding in liver cirrhosis. J Hepatol. (2000) 32:19–24. doi: 10.1016/s0168-8278(01)68827-5 10673062

[11] Zhao Y Ren MD Lu GF Lu XL Yin Y Zhang D . The prognosis analysis of liver cirrhosis with acute variceal bleeding and validation of current prognostic models: a large scale retrospective cohort study. Biomed Res Int. (2020) 2020:7372868. doi: 10.1155/2020/7372868, 32879889 PMC7448238

[12] Kumar AS Sibia RS. Predictors of in-hospital mortality among patients presenting with variceal gastrointestinal bleeding. Saudi J Gastroenterol. (2015) 21:43–6. doi: 10.4103/1319-3767.151226, 25672238 PMC4355862

[13] Elzouki AN Suliman S Alhasan R Abdullah A Othman M Badi A. Predicting mortality of patients with cirrhosis admitted to medical intensive care unit: an experience of a single tertiary center. Arab J Gastroenterol. (2016) 17:159–63. doi: 10.1016/j.ajg.2016.11.003, 27988236

[14] Con D Kemp W Majumdar A Pilcher D Roberts SK Majeed A. Long-term outcomes after ICU admission in critically ill patients with liver cirrhosis: an Australian state-wide cohort study. Hepatol Commun. (2025) 9:e0762. doi: 10.1097/hc9.0000000000000762 40689506 PMC12282839

[15] Bai Z Li B Lin S Liu B Li Y Zhu Q . Development and validation of CAGIB score for evaluating the prognosis of cirrhosis with acute gastrointestinal bleeding: a retrospective multicenter study. Adv Ther. (2019) 36:3211–20. doi: 10.1007/s12325-019-01083-5, 31512140 PMC6822790

[16] Bai Z Lin S Sun M Yuan S Marcondes MB Ma D . Machine learning based CAGIB score predicts in-hospital mortality of cirrhotic patients with acute gastrointestinal bleeding. NPJ Digit Med. (2025) 8:489. doi: 10.1038/s41746-025-01883-w, 40745090 PMC12313868

